# 2174 Building the next generation of translational researchers in health disparities

**DOI:** 10.1017/cts.2018.198

**Published:** 2018-11-21

**Authors:** Carlamarie Noboa, Zulmarie de Pedro-Serbía, Lourdes E. Soto de Laurido, Aracelis H. Chardon

**Affiliations:** University of Puerto Rico-Medical Sciences Campus

## Abstract

OBJECTIVES/SPECIFIC AIMS: Translational research involves researchers’ teams working together to address health issues. However, successful translational researchers in health disparities require a set of competencies and skills. In order to increase the number of new minority investigators in translational research focused on health disparities, the Hispanics-in-Research Capability: SoHP & SoM Partnership and the Puerto Rico Clinical and Translational Research Consortium designed and implemented a webinar series “Fostering the Next Generation of Researchers in Health Disparities.” METHODS/STUDY POPULATION: From March 31 to July 14, 2017, this webinar series offered the theoretical perspectives of health disparities, research methodology specific to its study, and intervention strategies to address health disparities in communities through minority investigators. National and local interdisciplinary experts were the presenters. Participants’ experience and impact were assessed through a self-administrated questionnaire. RESULTS/ANTICIPATED RESULTS: A total of 78 minority investigators participated in this webinar. Overall, participants indicated that the webinar improved their knowledge and skills about health disparities research. DISCUSSION/SIGNIFICANCE OF IMPACT: Results guide the programs actions plans to enhance and support the translational researchers’ capacity. Diverse capacity building initiatives including peer-to-peer education, online course, tailored coaching, and other interventions have been designed to address researchers’ needs. This webinar was a pathway to build the next generation of translational researchers in health disparities.

